# Fructooligosaccharide Supplementation Boosts Growth Performance, Antioxidant Status, and Cecal Microbiota Differently in Two Rabbit Breeds

**DOI:** 10.3390/ani12121528

**Published:** 2022-06-13

**Authors:** Ayman H. Abd El-Aziz, Mahmoud M. Abo Ghanima, Walaa F. Alsanie, Ahmed Gaber, Abd El-Wahab Alsenosy, Ahmed A. Easa, Sherif A. Moawed, Sayed Haidar Abbas Raza, Ahmed Elfadadny, Hany Abo Yossef, Wafaa M. Ghoneem, Mustafa Shukry, Amin Omar Hendawy, Khalid Mahrose

**Affiliations:** 1 Animal Husbandry and Animal Wealth Development Department, Faculty of Veterinary Medicine, Damanhour University, Damanhour 22511, Egypt; ayman.sadaka@vetmed.dmu.edu.eg (A.H.A.E.-A.); aboghoneima.mmyvet2@vetmed.dmu.edu.eg (M.M.A.G.); 2 Department of Clinical Laboratories Sciences, The Faculty of Applied Medical Sciences, Taif University, P.O. Box 11099, Taif 21944, Saudi Arabia; w.alsanie@tu.edu.sa; 3 Centre of Biomedical Sciences Research (CBSR), Deanship of Scientific Research, Taif University, P.O. Box 11099, Taif 21944, Saudi Arabia; a.gaber@tu.edu.sa; 4 Department of Biology, College of Science, Taif University, P.O. Box 11099, Taif 21944, Saudi Arabia; 5 Biochemistry Department, Faculty of Veterinary Medicine, Damanhour University, Damanhour 22511, Egypt; dr_alsenosy_2010@yahoo.com; 6 Department of Animal and Poultry Production, Faculty of Agriculture, Damanhour University, Damanhour 22516, Egypt; ahmed.allam@agr.dmu.edu.eg (A.A.E.); amin.hendawy@gmail.com (A.O.H.); 7 Department of Animal Wealth Development, Biostatistics Division, Faculty of Veterinary Medicine, Suez Canal University, Ismailia 41522, Egypt; sherifmoawed@vet.suez.edu.eg; 8 College of Animal Science and Technology, Northwest A&F University, Xianyang 712100, China; haiderraza110@nwafu.edu.cn; 9 Department of Animal Internal Medicine, Faculty of Veterinary Medicine, Damanhour University, Damanhour 22511, Egypt; ahmed.elfadadny@vetmed.dmu.edu.eg; 10 Food Hygiene Department, Faculty of Veterinary Medicine, Alexandria University, Edfina 22758, Egypt; Hanyyossef84@gmail.com; 11 Animal Production Department, Faculty of Agriculture, Cairo University, Giza 12613, Egypt; wafaaghoneem@agr.cu.edu.eg; 12 Department of Physiology, Faculty of Veterinary Medicine, Kafrelsheikh University, Kafrelsheikh 22511, Egypt; 13 Animal and Poultry Production Department, Faculty of Technology and Development, Zagazig University, Zagazig 44511, Egypt; ostrichkhalid@zu.edu.eg

**Keywords:** antioxidant status, carcass, fructooligosaccharide, growth, haemato-biochemical parameters, cecal microbiota

## Abstract

**Simple Summary:**

Rapidly rising incomes are dependent on animal protein production and the worldwide demand for livestock. It is expected that moving towards more intensive production systems to sustain this increased demand will depend on growth promoters. Some growth promoters, such as prebiotics, might be considered alternative non-antibiotic feed supplementation as they enhance performance without any side effects on the consumer’s health. The present study inspected the influence of supplementation of β-fructan^®^ (a commercial fructooligosaccharide; FOS) in the drinking water of growing rabbits on growth performance, carcass traits, hematological and biochemical indices, antioxidant status, and cecal microbiota of the NZW- and APRI-line rabbits (Animal Production Research Institute Line). FOS supplementation in rabbits enhanced growth carcass characteristics, significantly improving hematological parameters and antioxidant status, and minimized pathogenic Escherichia coli bacteria (from 3.45 in control groups to 2.89 and 2.24 (Log10 CFU g^−1^) in 0.5 mL and 1 mL FOS-treated rabbits, respectively.

**Abstract:**

The present study examined the effects of fructooligosaccharide (FOS) supplementation in drinking water on the growth performance, carcass characteristics, hematological and biochemical parameters, antioxidant status, and cecal microbiota of New Zealand White (NZW) and APRI rabbits. A total of 180 male NZW and APRI rabbits (aged five weeks; average live body weight 700 ± 39 g) were divided into six groups (30 rabbits/group; 5 replicates/group) in a two × three factorial arrangement. Rabbits of each breed were randomly assigned to one of three treatments of FOS (control; 0.00, FOS-0.5, and FOS-1.0). Results showed that rabbits’ final body weight, FBWG, and carcass traits were considerably enhanced compared to those in the control group. The interaction effect of the supplement with the rabbit breed increased the growth, carcass traits, and hematobiochemical and antioxidant parameters with increasing FOS levels. In the cecum of both rabbit breeds, the total bacterial count and *Escherichia* *coli* population were considerably low, with a substantial increase in the number of *Lactobacilli* supplemented by FOS. In conclusion, FOS supplementation enhanced growth and carcass traits by improving the hematobiochemical parameters and antioxidant status and reducing cecal pathogenic bacteria in both breeds.

## 1. Introduction

Improving animal productivity and boosting immunity using natural substances is a primary goal in animal breeding [1,2,3,4,5,6,7]. Recent studies have shown that immunostimulants, such as probiotics and prebiotics, have the potential to be used as protective and environment-friendly substitutions to antibiotics in mammals and poultry species [8,9,10,11]. These compounds are a possible method to enhance animal health and performance without antibiotics [12]. Prebiotics, such as inulin-type fructans and galacto-oligosaccharides, reveal immune-stimulating properties to the host through selective promoting of growth and/or encouraging the growth of some beneficial bacteria (i.e., probiotics) [13,14]. Fructooligosaccharides (FOSs) are considered the popular forms of prebiotics that consist of short-chain and undigested carbohydrates [9,15] because the β-linkages between fructose monomers cannot be hydrolyzed by the endogenous enzymes [16]. FOS is derived from the cell wall of the yeast, *Saccharomyces cerevisiae*, and has been reported to possess the ability to improve growth performance, decrease pathogenic bacterial count, and enhance immunity in two different rabbit breeds (New Zealand White and V-line rabbits) [1].

FOS may accelerate the gut fermentation of beneficial microorganisms, such as *Lactobacillus* and *Bifidobacterium*, and reduce the accumulation of pathogenic bacteria, such as *Clostridium perfringens* and *Escherichia coli* [15,17,18,19], thus enhancing the general health of animals [1,6,17]. Therefore, FOS is considered to be a prebiotic [20]. Dietary FOSs was reported to improve the growth traits (body weight, weight gain, and feed conversion ratio) and immune responses of broilers [21,22,23].

To our knowledge, there are no reports on adding FOSs to the drinking water of growing rabbits. Hence, this study was conducted to detect the possible effects of adding FOS (β-fructan^®^, a commercial FOS) in drinking water on the productive performance, carcass characteristics, hematobiochemical parameters, oxidative stress, and cecal microbiota of New Zealand White (NZW) and APRI rabbits. We hypothesized that oral FOS supplementation in combination with rabbit breed would enhance growth, improve blood biochemistry and antioxidant status, and improve microflora population diversity to alleviate the weaning stress of the rabbits.

## 2. Materials and Methods

### 2.1. Ethical Declaration

This research was performed after the approval of the Ethics of the Institutional Committee of Animal Husbandry and Animal Wealth Development Department, Faculty of Veterinary Medicine, Damanhour University, Egypt (DMU/VetMed-2019-/0145).

### 2.2. Animal Rearing and Study Design

APRI rabbit was produced by crossing Baladi Red bucks with a V line to create F1 (½B½V) stock, and it was continued for two generations of intersex mating to attain performing constancy. A total of 180 weaned APRI and NZW rabbits (male, aged five weeks, weighing 700 ± 39 g) were collected and allocated to six groups (30 rabbits per group), and each group was divided into five replicates, each with six rabbits. The rabbits were assigned at random using a two × three completely factorial design (NZW and APRI-line with three treatments of a commercial FOS known as β-fructan^®^). The control group was not treated with FOS, and the first and second groups were supplemented with FOS-0.5 mL and FOS-0.1 mL, respectively. The experimental groups received 0.5 and 1.0 mL β-fructan (1,3 pharmaceutical grade 10%) per liter of drinking water for three sequential days per week (Glencore Company, Ann Arbor, MI, USA). Each rabbit in the 0.5 mL β-fructan-treated group was supplemented with 349.8 mg of β-fructan during the eight-weeks experimental period, while in the 1 mL β-fructan-treated group, each rabbit was supplemented with 699.75 mg of β-fructan. Rabbits were housed in galvanized wire batteries with standard dimensions (60 × 35 × 35 cm). All cages were supplied with galvanized steel feeding hoppers and automatic drinkers (nipples). Plastic ear tags identified rabbits. Freshwater was provided *ad libitum*, and a standard pelleted ration was provided *ad libitum* twice daily at 8 am and 2 pm. The pellets measured 1 cm in length and 0.4 cm in diameter. Rabbit cages were regularly cleaned and disinfected. Urine and feces dropped beneath the batteries were removed every morning.

### 2.3. Experimental Feed Diet Preparation

Diet was prepared following the NRC [24] and Lebas [25] recommendations (Table 1). The analysis of the ingredients was performed according to AOAC [26].

### 2.4. Productive Performance and Carcass Characteristics

At the start of the fifth week, the animals were weighed individually until the end of the experiment (13 weeks of age). The rabbit’s daily feed consumption was calculated every week to evaluate the feed conversion ratio (FCR). Final body weight (FBW), body weight gain (BWG), and total feed consumption (TFC) were determined. Fifteen rabbits from each group were randomly selected to evaluate carcass characteristics at the end of the experiment (13th week). Rabbits were fasted for 12 h before being slaughtered. After removing the skin and bones, the carcasses were measured individually to evaluate the weight and percentage of the dressed animals. The offal weight includes blood, viscera, lungs, skin, arms, and tail. The obtained results were presented as the % of live weight [27]. The dressing % was calculated as hot carcass weight × 100/fasting weight. The carcass was divided into three cuts, viz., (1) the two forelegs (including the thoracic muscle inserting system), (2) the loin (the abdominal wall and the riveting after the seventh thoracic rib), and (3) the hind legs (including the sacral bone and the lumbar vertebra after the sixth lumbar vertebra).

### 2.5. Hematology and Biochemical and Serum Oxidative Stress Evaluations

Two blood samples were collected from the lateral ear vein (30 rabbits) during the slaughter. One sample contained an anticoagulant and was used to determine the count of white blood cells (WBCs), red blood cells (RBCs), lymphocytes, monocytes, and mean corpuscular hemoglobin (MCH), red cell distribution width (RDW), platelet count, hematocrit %, and hemoglobin concentration [28]. The other blood sample was centrifuged (15 min, 3000× *g*) at 15–24 °C for plasma separation and stored at −20 °C until analysis. Total protein, albumin, cholesterol, alanine aminotransferase (ALT), aspartate aminotransferase (AST), and creatinine levels were measured in plasma using commercial kits. Moreover, the levels of glutathione peroxidase (GPX), superoxide dismutase (SOD), and total antioxidant capacity (T-AOC) were evaluated using the colorimetric method (kits obtained from Bio-diagnostic, Cairo, Egypt).

### 2.6. Bacterial Count

Total bacteria, *E. coli*, and *lactobacilli* were all counted using the ring-plate method in the rabbit cecum sample [29,30].

### 2.7. Data Analysis

The attained results were statistically analyzed with the general linear model procedure of SAS^®^ (Cary, NC, USA) [31]. Homogeneity of variances among studied groups was verified [32]. The analysis was performed using this model: Y_ijk_ = μ + S_i_ + E_j_ + SE_ij_ + e_ijK_, where μ = observed mean for the concerned treatment, S_i_ = breed effect, E_j_ = treatment effect, SE_ij_ = interaction effect of breed and treatment, and e_ijk_ = the error related to individual observation using Duncan’s multiple range test [32]. According to Ahrens et al. [33], the percentages were converted into arcsine values. Results were considered statistically significant at *p* ≤ 0.05.

## 3. Results

The FBW, FBWG, TFC, and FCR of rabbits supplemented with oral FOS were considerably enhanced compared to those in the control group (Table 2). The NZW rabbits treated with 1% FOS in drinking water showed the largest FBW, followed by APRI rabbits in which drunk water increased with the same level of FOS. BWG was more significant in NZW rabbits that consumed 1% and 0.5% FOS and APRI rabbits that consumed water supplemented with 1% FOS than their counterparts. Increasing FOS concentrations in drinking water decreased (*p* < 0.001) the amount of feed consumed in both breeds. The NZW rabbits that consumed water supplemented with 1% and 0.5% FOS and the APRI rabbits that consumed 1% FOS-supplemented water showed the lowest (*p* < 0.001) FCR compared with the other groups (Table 2).

Total giblets, gastrointestinal tract, liver, and dressing % in NZW and APRI rabbits that consumed FOS-supplemented water were significantly enhanced due to FOS and the interaction between FOS and breed (*p* < 0.001) compared with the control groups (Table 3). The difference between FOS levels was insignificant.

Substantial improvements (*p* < 0.001) were found in the two breeds in hematobiochemical and antioxidant parameters (Table 4 and Table 5), which were enhanced with increasing FOS levels (FOS-1.0), with no detrimental effects on the kidney and liver. However, rabbits consuming 1% FOS had higher blood biochemicals and antioxidant parameters values than those receiving 0.5% FOS-supplemented water.

In both rabbit breeds that consumed FOS-supplemented water, the cecum, total bacterial, and *E. coli* populations (Table 6) were considerably lower (*p* < 0.001), with a substantial increase in the Lactobacillus population compared with the control groups. Rabbits that consumed 1% FOS showed the most significant count of beneficial bacteria and a lower count of pathogenic ones compared with the other treatments.

## 4. Discussion

This study investigated the possible effects of adding FOS in drinking water on the growth performance, carcass characteristics, hematobiochemical parameters, oxidative stress biomarkers, and cecal microbiota of NZW and APRI rabbits. Our results showed that supplementing water with FOS significantly enhanced the growth performance traits of the two rabbit breeds.

The beneficial effects of adding FOS to the drinking water of growing rabbits may be due to the augmentation of feed efficiency and absorption, which improves anabolic metabolism, enhances the intestinal response to pathogens, and increases serum protein levels, thereby encouraging rabbit growth [1,6,7]. Prebiotics provide suitable environments for the growth of helpful microflora and inhibit the growth of pathogenic bacteria, which may explain the improvement in growth performance [7].

Consistent with our findings, the rabbits’ growth was enhanced with *Bacillus subtilis* and FOS with a more significant average daily BWG than the control [22]. In addition, Inmunair17.5^®^ (*Propionibacterium acnes* and coli lipopolysaccharides) as a prebiotic in the drinking water of fattening NZW rabbits resulted in an enhancement of BW at marketing, BWG, and FCR [11]. Comparable findings reported that a diet supplemented with *S.* *cerevisiae* and probiotics accelerated the BWG and FCR of NZW rabbits [34,35]. By contrast, Rotolo et al. [36,37] found that the dietary supplementation of *S.* *cerevisiae* did not affect rabbits’ BW, BWG, and FCR. Additionally, Zarei et al. [38] reported that dietary prebiotics did not modify FCR in laying hens. In broilers, Xu et al. [14] concluded that supplementation with 4 g of FOS/kg diet increased BWG and improved FCR.

Regarding carcass characteristics, our findings were consistent with those observed by Abd El-Aziz et al. [1], Mahrose et al. [6], and Abo Ghanima et al. [2]. Similarly, Mousa et al. [11] showed that dressing and giblet percentages were significantly higher in the carcasses of rabbits that drank water supplemented with 1 mL Inmunair17.5^®^/litter. However, Rotolo et al. [36] found nonsignificant changes in the carcass characteristics of growing rabbits treated with dietary prebiotics. Moreover, Juśkiewicz et al. [39] concluded that increasing turkeys fed with a diet supplemented with FOS showed no differences from the control group. There were no significant changes between the two rabbit breeds regarding the breed impact on carcass traits in the present study. Such an absence of significant differences in carcass traits between genetic breeds has also been confirmed in previous studies [1,7].

Hematological measurements are valuable indicators for evaluating the animals’ health statuses [4]. In our study, most hematological parameters were altered by the water supplemented with FOS in the two rabbit breeds. Our findings are consistent with those of Akrami et al. [13], who found that WBC counts were increased in fish fed with 1% FOS compared with the control group. They also found a nonsignificant elevation of RBCs, MCV, HCT, Hgb, and lymphocytes in the fish fed with a diet supplemented with 1% FOS.

In a study on birds, FOS supplementation resulted in low heterophil counts, indicating that FOS may reduce stress reactions and alleviate the possible damaging consequences on growth performance [9]. Moreover, broilers supplemented with FOS had more significant monocyte counts than broilers fed with the control diet. Monocytes comprise 5%–10% of peripheral blood leukocytes and can migrate rapidly in response to diseases, release cytokines, and differentiate into macrophages and dendritic cells to assist the innate immune response [40]. FOS supplementation increased monocyte %, suggesting that dietary FOS supplementation in broilers augments cytokine release and alleviates pathogenic infections rapidly [9]. This effect is probably due to the alteration in the gut microbiota, such as variations in the *Lactobacillus* profile, which shows diverse patterns for dendritic cell activation [41,42]. The findings concerning hematological indices revealed that these measurements were increased in rabbits that consumed water supplemented with FOS-0.5 and FOS-0.1. Hoseinifar et al. [43] mentioned that WBC count, primarily lymphocytes, was significantly increased in belugas fed with 1 and 2 g kg^−1^ dietary oligofructose. The high leukocyte count may increase activity and improve defense mechanisms during feeding. Leukocytes are imperative cells that stimulate the immune responses of fish. They produce antibodies and may exhibit macrophage activities [44]. Saha et al. [19] obtained similar results, where the MCH in broilers receiving a water-soluble organic additive at different doses fluctuated from that in the control.

The total protein and globulin levels were increased in the experimental groups treated with varying levels of FOS in their diets, indicating a more robust innate immune response. Globulin is believed to be the main protein that plays a significant role in immune response [5]. Moreover, FOS was found to have the potential to control enteric pathogens and alter immunity [1]. This result was also previously supported by Abd El-Gawad et al. [17] who concluded that ALT and AST activities were diminished with dietary FOS than in the control fish group. Our results failed to show significant differences in AST and ALT activities with FOS supplementation in drinking water.

Interestingly, our findings showed an increase in SOD, GPX, and T-AOC values in the supplemented groups of the two rabbit breeds. These results suggest that the FOS-supplemented drinking water could alleviate oxidative stress in the two breeds of growing rabbits and maintain their healthy. The first line of antioxidant enzymatic defense is believed to involve GPX, SOD, and T-AOC [5], which act as biomarkers of oxidative stress due to the inequality between the production and elimination of reactive oxygen species. The enhancement of antioxidant enzymatic activities in the present study with FOS supplementation in the drinking water of growing rabbits was also previously reported by Guerreiro et al. [45] and Zhang et al. [46] as FOS supplementation may relieve oxidative stress [17].

In the present study, FOS supplementation in drinking water caused a stimulatory impact on the growth of health-supporting bacterial species (*Lactobacillus*). Moreover, FOS supplementation decreased the total bacterial count and harmful or potential pathogens (*E. coli*) in the two rabbit breeds. Our results are consistent with those reported by Xu et al. [16] who examined the effects of FOS at doses of 0, 2, 4, and 8 g/kg diet on intestinal microbiota. The inclusion of FOS at a 4 g/kg diet resulted in a beneficial effect on *Bifidobacterium* and *Lactobacillus*, with an immediate reduction of *E. coli* growth in the broilers’ gastrointestinal tract. Saminathan et al. [47] evaluated the impact of applying various oligosaccharides by isolating 11 *Lactobacillus* species from the gastrointestinal tract of fowls. The in vitro data revealed that Lactobacillus species utilized FOS more competently than mannan oligosaccharides. The increased availability of FOS may be related to particular enzymatic actions and the oligosaccharide conveyance technique of *Lactobacillus* species. Nevertheless, broilers’ intestinal microbiota is further complicated than in vitro examinations. Prebiotics may be fermented not only by *Lactobacillus* species but also by other microbes in the gastrointestinal tracts of animals [23].

## 5. Conclusions

FOS supplementation in the drinking water of rabbits improved most growth performance parameters, carcass characteristics, hematobiochemical parameters, antioxidant status, and cecal microbiota in NZW and APRI rabbits. Moreover, the response of NZW rabbits to FOS supplementation was more significant than that of APRI rabbits.

## Figures and Tables

**Table 1:** Ingredients and chemical composition (%) of the basal diet.

Ingredients	%
Yellow corn	9.5
Soybean meal (44%)	15
Wheat bran	17
Barley	21.7
Barley hay	34.5
Dicalcium phosphate ^1^	1.2
Ground limestone ^2^	0.25
DL-Methionine	0.05
Common salt	0.5
Vitamin + mineral premix ^3^	0.3
Total	100
Chemical composition	
Dry matter	87.8
Moisture	12.2
Crude protein	17.9
Crude fiber	13.75
Ether extract	3.6
Nitrogen-free extract ^4^	42.75
Ash	9.8
DE (kcal /kg) ^5^	2677.97

^1^ Dicalcium phosphate: 20% phosphorus and 25% calcium; ^2^ limestone: 34% calcium. ^3^ Amounts per kg: Vitamin A—12,000 and 900 IU of vitamin A and D3, respectively. While 2 mg of each vitamin K3, B1, and B6. 50 mg of vitamin E, 6 mg vitamin B2, 0.01 mg vitamin B12, 0.2 mg biotin, 20 mg pantothenic, 50 mg niacin, 5 mg folic acid, 8.5 mg manganese, 70 mg zinc, 75 mg iron, 5 mg copper, 0.75 mg iodine, 0.1 mg selenium. ^4^ Nitrogen free extract (NFE) was calculated by difference = 100 − (moisture % + CP% + EE% + CF% + Ash %). ^5^ Digestible energy (DE) was calculated according to values given in the feed composition tables of the NRC [24].

**Table 2:** Growth performance of rabbits as affected by breed and supplementation of the diets with fructooligosaccharide (FOS).

Items		Initial Body Weight (g)	Final Body Weight (g)	Body Weight Gain (g)	Total Feed Consumption (g)	Feed Conversion Ratio (g Feed/g Gain)
Breed						
NZW		756.45	2261.45	1464.50	4740.24	3.278
APRI		706.25	2207.91	1461.54	4736.51	3.281
FOS supplementation						
FOS 0.5 mL/L DW		721.25	2160.62	1470.00	4647.45	3.19
FOS 1 mL/L DW		737.18	2409.06	1561.56	4609.54	2.96
Control		735.62	2134.37	1357.50	4958.14	3.68
Breed × Treatment interaction						
NZW	Control	768.12	2139.37 ^c^	1389.37 ^cd^	4914.37 ^b^	3.65 ^a^
	FOS 0.5 mL	738.12	2176.25 ^c^	1519.62 ^abc^	4699.77 ^c^	3.11 ^bc^
	FOS 1 mL	763.12	2500.00 ^a^	1583.75 ^a^	4606.58 ^d^	2.92 ^c^
APRI	Control	703.12	2129.37 ^c^	1325.62 ^d^	5001.91 ^a^	3.71 ^a^
	FOS 0.5 mL	704.37	2145.00 ^c^	1420.37 ^bcd^	4595.12 ^d^	3.26 ^b^
	FOS 1 mL	711.25	2318.12 ^b^	1539.37 ^ab^	4612.50 ^d^	3.007 ^bc^
SEM		18.383	19.472	18.249	8.151	0.044
Two-way ANOVA (*p*-value)						
Breed		0.179	0.176	0.936	0.820	0.979
Treatment		0.927	˂0.001	˂0.001	˂0.001	˂0.001
Interaction		0.827	˂0.001	0.001	˂0.001	˂0.001

Means within each column for each division with no common superscript letters are significantly different (*p* ˂ 0.05). SEM = standard error of means.

**Table 3:** Carcass traits of rabbits as affected by breed and supplementation of the diets with fructooligosaccharide (FOS) (%).

Items		Forequarter	Loin	Hindquarter	Giblets	Gastrointestinal Tract	Liver	Dressing
Breed								
NZW		33.48	27.10	39.21	3.67 ^a^	25.59	5.04	55.24
APRI		33.28	27.01	39.19	3.20 ^b^	27.24	4.64	54.74
FOS supplementation								
FOS 0.5 mL/L DW		33.22	27.26	39.26	3.64 ^a^	26.38 ^ab^	5.02 ^a^	55.18 ^a^
FOS 1 mL/L DW		33.73	27.60	39.68	3.71 ^a^	24.13 ^b^	5.17 ^a^	56.61 ^a^
Control		33.20	26.32	38.66	2.97 ^b^	28.73 ^a^	4.34 ^b^	53.19 ^b^
Breed x Treatment interaction								
NZW	Control	33.16	26.93	38.43	3.11 ^b^	28.32 ^a^	4.15 ^b^	53.13 ^b^
	FOS 0.5 mL	33.28	27.38	39.74	3.28 ^b^	25.77 ^a^	4.92 ^ab^	56.28 ^a^
	FOS 1 mL	34.23	27.81	40.08	4.20 ^a^	21.86 ^b^	5.67^a^	56.93 ^a^
APRI	Control	32.23	25.72	37.87	2.66 ^b^	29.14 ^a^	4.15 ^b^	53.06^b^
	FOS 0.5 mL	33.23	27.01	39.43	3.21 ^b^	26.99 ^a^	4.53 ^ab^	53.98 ^b^
	FOS 1 mL	34.06	27.51	39.62	4.16 ^a^	26.39 ^a^	5.12 ^ab^	56.37 ^a^
SEM		0.214	0.250	0.243	0.081	0.490	0.128	0.269
Two-way ANOVA (*p-value*)								
Breed		0.645	0.850	0.976	0.010	0.110	0.137	0.413
Treatment		0.350	0.127	0.255	0.003	0.005	0.038	0.001
Interaction		0.185	0.262	0.119	˂0.001	0.008	0.047	0.003

Means within each column for each division with no common superscript letters are significantly different (*p* ˂ 0.05).

**Table 4:** Hematological parameters of rabbits as affected by breed and supplementation of the diets with fructooligosaccharide (FOS).

Item		WBC 10^3^/µL	Lymphocytes 10^3^/µL	Monocytes 10^3^/µL	RBC 10^6^/µL	Hgb %	MCV ft	HCT %	MCH pg	RDW %	Platelets 10^3^/µL
Breed											
NZW		6.11	4.36 ^a^	0.59	4.47	11.07	60.88	32.22	24.39	25.37 ^a^	152.07
APRI		5.94	3.71 ^b^	0.58	4.41	11.06	61.60	34.83	24.80	24.95 ^b^	153.07
FOS supplementation											
FOS 0.5 mL/L DW		5.95 ^b^	3.62 ^b^	0.57 ^b^	4.46	11.12 ^a^	61.55 ^b^	34.55 ^ab^	24.90 ^a^	25.27 ^b^	153.60 ^b^
FOS 1 mL/L DW		7.57 ^a^	5.81 ^a^	0.65 ^a^	4.44	11.24 ^a^	64.53 ^a^	36.46 ^a^	25.319 ^a^	26.52 ^a^	160.40 ^a^
Control		4.56 ^c^	2.69 ^c^	0.53 ^c^	4.43	10.83 ^b^	57.65 ^c^	29.57 ^b^	23.70 ^b^	23.69 ^c^	143.70 ^c^
Breed × Treatment interaction											
NZW	Control	4.72 ^e^	3 ^d^	0.532 ^c^	4.42	10.84 ^b^	58.18 ^d^	33.12 ^ab^	24 ^ab^	23.80 ^d^	144.80 ^c^
	FOS 0.5 mL	6.12 ^c^	3.71 ^c^	0.586 ^b^	4.44	11.14 ^a^	62.24 ^bc^	34.96 ^a^	24.98 ^ab^	25.40 ^c^	153.80 ^b^
	FOS 1 mL	7.83 ^a^	6.38 ^a^	0.664 ^a^	4.49	11.24 ^a^	64.68 ^a^	36.50 ^a^	25.40 ^a^	26.90 ^a^	160.60 ^a^
APRI	Control	5.78 ^d^	2.38 ^e^	0.528 ^c^	4.44	10.82 ^b^	57.12 ^d^	26.02 ^b^	23.40 ^b^	23.58 ^d^	142.60 ^c^
	FOS 0.5 mL	7.32 ^b^	3.52 ^c^	0.566 ^b^	4.42	11.10 ^a^	60.86 ^c^	34.14 ^a^	24.80 ^ab^	25.14 ^c^	153.40 ^b^
	FOS 1 mL	7.32 ^b^	5.24 ^b^	0.646 ^a^	4.49	11.24 ^a^	64.38 ^ab^	36.42 ^a^	25 ^ab^	26.14 ^b c^	160.20 ^a^
SEM		0.047	0.047	0.004	0.037	0.024	0.324	1.05	0.215	0.084	0.840

Breed		0.101	0.001	0.205	0.380	0.891	0.281	0.226	0.355	0.021	0.557
Treatment		0.001	0.001	0.001	0.958	0.001	0.001	0.036	0.022	0.001	0.001
Interaction		0.001	0.001	0.001	0.951	0.001	0.001	0.077	0.115	0.001	0.001

Means within each column for each division with no common superscript letters are significantly different (*p* ˂ 0.05). SEM: standard error of the means; MCV: mean corpuscular volume; HCT; hematocrit; MCH: mean corpuscular hemoglobin; RDW: red cell distribution width.

**Table 5:** Blood biochemical parameters and some selected oxidative stress biomarkers of rabbits as affected by breed and supplementation with fructooligosaccharide (FOS).

Item		Serum Biochemical Parameters								Oxidative Stress Biomarkers		
		Total Protein (g/dL)	Albumin (g/dL)	Globulin (g/dL)	A/G Ratio	Cholesterol (mg/dL)	ALT (U/L)	AST (U/L)	Creatinine (mg/dL)	GPX (U/L)	SOD (U/L)	T-AOC (mmol/L)
Breed												
NZW		6.45	3.70	2.75	1.48	50.80	35	31.87	1.74	27.27 ^a^	79.40	1.32
APRI		6.42	3.69	2.72	1.43	54.80	35.66	31.93	1.74	25.80 ^b^	78.60	1.31
FOS supplementation												
FOS 0.5 mL/L DW		6.59 ^b^	3.69	2.89 ^b^	1.28 ^b^	48 ^b^	35.10	32.60	1.79	27.70 ^b^	80.30 ^b^	1.33 ^b^
FOS 1 mL/L DW		7.14 ^a^	3.74	3.39 ^a^	1.11 ^b^	33.30 ^c^	36.10	31.40	1.69	31.40 ^a^	88.40 ^a^	1.36 ^a^
Control		5.57 ^c^	3.65	1.91 ^c^	1.98 ^a^	77.10 ^a^	34.80	31.70	1.74	20.50 ^c^	68.30 ^c^	1.27 ^c^
Breed × Treatment interaction												
NZW	Control	5.60 ^c^	3.67	1.97 ^c^	1.87 ^a^	76.6 ^a^	34.4	31.6	1.74	20.80 ^d^	68.60 ^c^	1.28 ^c^
	FOS 0.5 mL	6.60 ^b^	3.70	2.90 ^b^	1.27 ^b^	44 ^c^	34.8	32.6	1.73	28.20 ^bc^	81.40 ^b^	1.33 ^b^
	FOS 1 mL	7.20 ^a^	3.78	3.50 ^a^	1.06 ^b^	31.80 ^d^	35.8	31.4	1.67	32.80 ^a^	88.60 ^a^	1.36 ^a^
APRI	Control	5.54 ^c^	3.62	1.85 ^c^	2.09 ^a^	77.6 ^a^	35.2	31.8	1.81	20.20 ^d^	68 ^c^	1.27^c^
	FOS 0.5 mL	6.58 ^b^	3.68	2.89 ^b^	1.28 ^b^	52 ^b^	35.4	32.7	1.78	27.20 ^c^	79.20 ^b^	1.33 ^b^
	FOS 1 mL	7.08 ^a^	3.71	3.29 ^ab^	1.15 ^b^	34.80 ^d^	36.4	31.4	1.71	30 ^b^	88.20 ^a^	1.34 ^ab^
SEM		0.049	0.020	0.054	0.048	1.018	0.293	0.374	0.023	0.248	0.728	0.004
Two-way ANOVA (*p-value*)												
Breed		0.789	0.960	0.774	0.638	0.061	0.257	0.930	0.989	0.016	0.588	0.518
Treatment		0.001	0.221	0.001	0.001	0.001	0.188	0.409	0.183	0.001	0.001	0.001
Interaction		0.001	0.367	0.001	0.001	0.001	0.450	0.860	0.571	0.001	0.001	0.001

Means within each column for each division with no common superscript letters are significantly different (*p* ˂ 0.05). SEM: standard error of the means; ALT; alanine aminotransferase; AST; aspartate aminotransferase; GPX, glutathione peroxidases; SOD, superoxide dismutase; T-AOC, total antioxidant capacity.

**Table 6:** Cecal bacterial counts of rabbits as affected by breed and supplementation with fructooligosaccharide (FOS) (Log10 CFU g^−1^).

Items		Total Bacterial Count (TBC)	*Cecal Escherichia coli* (*E. coli*)	*Cecal Lactobacilli*
Breed				
NZW		7.09	2.82 ^b^	7.57
APRI		7.14	2.96 ^a^	7.60
FOS supplementation				
FOS 0.5 mL/L DW		7.03 ^b^	2.89 ^b^	7.86 ^a^
FOS 1 mL/L DW		6.05 ^c^	2.24 ^c^	7.95 ^a^
Control		8.26 ^a^	3.54 ^a^	6.94 ^b^
Breed x Treatment interaction				
NZW	Control	8.24 ^a^	3.50 ^a^	6.90 ^b^
	FOS 0.5 mL	7 ^b^	2.84 ^b^	7.80 ^a^
	FOS 1 mL	5.98 ^c^	2.12 ^d^	7.92 ^a^
APRI	Control	8.28 ^a^	3.58 ^a^	6.98 ^b^
	FOS 0.5 mL	7.06 ^b^	2.94 ^b^	7.90 ^a^
	FOS 1 mL	6.13 ^c^	2.36 ^c^	8 ^a^
SEM		0.027	0.029	0.081
Two-way ANOVA (*p-value*)				
Breed		0.697	0.024	0.839
Treatment		˂0.001	˂0.001	0.001
Interaction		˂0.001	˂0.001	0.001

Means within each column for each division with no common superscript letters are significantly different (*p* ˂ 0.05). SEM = standard error of means.

## Data Availability

The data that support the findings of this study are available on request from the corresponding author. The data are not publicly available due to privacy or ethical restrictions.
